# ACE1 does not influence cerebral Aβ degradation or amyloid plaque accumulation in 5XFAD mice

**DOI:** 10.1371/journal.pone.0330193

**Published:** 2025-09-15

**Authors:** Sohee Jeon, Alia O. Alia, Jelena Popovic, Robert Vassar, Leah K. Cuddy

**Affiliations:** 1 The Ken and Ruth Davee Department of Neurology, Northwestern University Feinberg School of Medicine, Chicago, Illinois, United States of America; 2 Mesulam Center for Cognitive Neurology and Alzheimer’s Disease, Northwestern University Feinberg School of Medicine, Chicago, Illinois, United States of America; University of Tartu, ESTONIA

## Abstract

Alzheimer’s disease is the most common form of dementia, and multiple lines of evidence support the relevance of Aβ deposition and amyloid plaque accumulation in the neurotoxicity and cognitive decline in AD. Rare mutations in angiotensin-converting-enzyme-1 have been highly associated with late onset AD patients; however, the mechanism for ACE1 mutation in AD pathogenesis is unknown. While numerous studies have shown that ACE1 indeed catabolizes Aβ, majority of these studies were performed *in vitro,* and conflicting results have been reported in clinical and *in vivo* systems. Therefore, we further investigated this *in vivo* by generating and examining a novel mouse model. Specifically, we analyzed 6-month-old 5XFAD mice with ACE1 knockdown restricted to excitatory neurons, achieved by driving Cre recombinase expression under the CamKIIα promoter. These mice were generated by crossing 5XFAD mice to ACE1 conditional knockout mice expressing Cre specifically in excitatory neurons. Our analyses revealed that neuronal ACE1 knockdown does not significantly affect amyloid plaque load and neuroinflammation in the hippocampus and cortex of 5XFAD mice at 6-months of age.

## Introduction

Alzheimer’s disease (AD) is a neurodegenerative disorder that affects around 6.9 million Americans ages 65 and older in the United States [1]. AD initially manifests as a decline in memory and cognition, and eventually, leads to death [1,2]. Distinct from other forms of dementia, the pathological hallmarks of AD consist of amyloid plaques and neurofibrillary tangles. Specifically, amyloid plaques consist primarily of amyloid beta peptides (Aβ), particularly the neurotoxic Aβ42, which initially accumulates in neurons, and subsequently aggregates extracellularly in the cerebral cortex, subiculum, hippocampus, and other regions of the brain as the disease progresses [3–5]. Multiple lines of evidence support the relevance of Aβ deposition and amyloid plaque accumulation in the neurotoxicity and cognitive decline in AD [4,6,7]. Furthermore, amyloid plaque accumulation has been linked to mutations in APP, PSEN1, and PSEN2; these mutations lead to familial autosomal dominant early onset AD (EOAD) which comprise less than 5% of AD cases [7,8]. Symptomatically indistinguishable from EOAD, late onset AD (LOAD) comprises most AD cases and associates with mutations such as APOE and TREM2 [3].

Genome-wide association studies (GWAS) on AD patients revealed additional genetic mutations highly associated with LOAD, including those in ACE1 [9,10]. ACE1 encodes for angiotensin I converting enyme, an enzyme that cleaves angiotensin I (AngI) to angiotensin II (AngII) for activation of various receptors, including AngII receptor type 1 (AT1R) and AngII receptor type 2 (AT2R), in the renin-angiotensin-system (RAS) [11,12]. Within the brain, ACE1 is expressed in the brainstem, cerebellum, cortex, and hippocampus, and it is mostly expressed in neurons, specifically in the CA region excitatory neurons [10,13]. While the peripheral RAS has been well known for its role in blood pressure control, the central RAS has been linked to neurocognition [12,14–16]. Furthermore, most analyses of postmortem brain, cerebral spinal fluid (CSF), and plasma from AD patients reveal alterations in ACE1 level and/or activity, further suggesting a connection between AD and ACE1 [9,10,17,18]. In particular, our previous study identified the ACE1 R1279Q mutation in AD families through whole genome sequencing, and its cognate mutation in knock-in (KI) mice caused age-dependent brain atrophy and hippocampal neurodegeneration [10]. This study further examined 5XFAD mice with the ACE1 KI mutation and reported that, while Aβ accelerated neurodegeneration, Aβ42 levels remained unchanged in these mice [10]. Given the relevance of ACE1 with AD and the strong association of Aβ to AD pathogenesis, multiple previous studies sought to understand whether ACE1 affects Aβ deposition and amyloid accumulation.

Past studies investigating the role of ACE1 in AD suggest that ACE1 cleaves and degrades Aβ [19,20]. Although few *in vivo* studies support the hypothesis that ACE1 contributes to Aβ degradation, most *in vitro* studies have reported that the N-domain of ACE1 specifically cleaves Aβ [21–25]. While such studies imply ACE1’s role in Aβ cleavage and degradation [20,26–28], most clinical and *in vivo* data suggest otherwise [18,29]. For example, several clinical studies suggest that administration of centrally acting ACE1 inhibitors to AD patients delays or prevents cognitive decline, improves cognition, and reduces the progression of disease neuropathology; thus, inhibiting ACE1 may have clinical benefits [30–37]. Adding to this conundrum, several *in vivo* and clinical studies suggest that ACE1 does not affect Aβ levels in the brain and further report no significant difference in AD incidence with ACE1 inhibitors [38–45]. Altogether, these findings suggest conflicting results regarding the role of ACE1 in Aβ degradation. However, discrepancies may arise due to factors such as study design, the limited ability of ACE1 inhibitors to cross the blood-brain barrier and their effects on non-neuronal cells, despite ACE1 being predominantly expressed in neurons [10,13].

Here, we investigated whether ACE1 degrades Aβ and affects amyloid burden in the AD brain *in vivo*. We developed a novel mouse model where ACE1 is knocked down specifically in forebrain neurons of the 5XFAD mouse model of amyloidosis. Investigating whether genetic knockdown of neuronal ACE1 impacts Aβ burden is important to not only understand the mechanism of neuronal ACE1 in AD pathogenesis, but also to examine the neurocognitive effect of ACE inhibitors as a treatment for hypertensive patients. For this, we examined amyloid burden in 5XFAD amyloid transgenic mice with neuronal ACE1 loss of function, and we hypothesized that neuronal ACE1 does not impact Aβ levels *in vivo* based on previous transgenic AD mouse studies.

## Materials and methods

### Animals

All animal work was done in accordance with Northwestern University Institutional Animal Care and Use Committee (IACUC) approval, assurance number A3283-01. Mice were fed a standard rodent chow diet and water ad libitum and housed with a standard 12-hour light/dark cycle. 5XFAD mice on a C57BL6 background (RRID:MMRRC_034848-JAX) were maintained by crossing transgene positive hemizygous male C57BL6 mice with transgene negative female C57BL6 mice (RRID:IMSR_JAX:000664). To generate ACE1 conditional knockdown mice, the targeting strategy was based on NCBI transcript NM_207624.5 which corresponds to Ensembl transcript ENSMUST00000001963 (Ace-001). The positive selection marker (Puromycin resistance, PuroR) was flanked by FRT sites and was inserted into intron 13. The targeting vector was generated using BAC clones from the C57BL/6J RPCI-23 BAC library and transfected into the Taconic Biosciences C57BL/6N Tac ES cell line. Homologous recombinant clones were isolated using positive (PuroR) and negative (Thymidine kinase, Tk) selections. The conditional KO allele was obtained after in vivo Flp-mediated removal of the selection marker. Deletion of exons 14 and 15 resulted in the loss of function of the *Ace* gene by deleting part of the Extra Cellular Domain and by generating a frameshift from exon 13 to exons 16–18, a premature stop codon is in exon 16. The remaining recombination site is located in a non-conserved region of the genome. Cre driver mice that express cre-recombinase under the control of the CamKIIα promoter (RRID:IMSR_JAX:005359) were used to generate 5XFAD; ACE1^FL/FL^iCre mice with significantly reduced ACE1 protein levels specifically in the excitatory forebrain neurons. Transgene positive and negative ACE1^FL/+^ mice were crossed to ACE1^FL/+^ heterozygous CamKIIα-iCre mice to generate ACE1^FL/FL^ iCre mice, as well as ACE1^FL/FL^, ACE1^+/+^iCre and ACE1^+/+^ littermates, which served as controls for the study.

All animal experiments were conducted in accordance with the ARRIVE guidelines. Sample sizes were determined by power analysis to ensure sufficient statistical power. Animals were randomly assigned to experimental groups, and investigators were blinded to genotype and treatment during data collection and analysis. Both male and female mice were included in all experiments, and data were analyzed for sex-specific effects when possible. Mice were monitored daily and humane endpoints were established based on institutional guidelines. Inclusion criteria required animals of the correct genotype and age with no overt health issues; any exclusions and reasons were documented. All relevant data are provided within the manuscript and its Supporting Information files.

### Mouse brain extraction

Mice were measured for their body weight and were subsequently euthanized by intraperitoneal injection of ketamine (100 mg/kg) and xylazine (15 mg/kg). Then, mice were perfused with the Perfusion solution (20 µg/ml phenylmethyl sulfonyl fluoride, 0.1mM dithiothreitol, 0.5 µg/ml leupeptin, and 20µM sodium orthovanadate in 1x PBS). This was immediately followed by brain removal and brain weight measurement. Brains were divided in half along the sagittal plane for hemibrains which were immediately preserved for downstream experiments. Hemibrains from perfused mice were fixed in 10% formalin and preserved in 30% sucrose 1x PBS solution at 4^o^C. For experiments requiring analysis of specific brain regions, hemibrains were dissected on ice for the hippocampus, cortex and cerebellum and flash frozen in liquid nitrogen for storage at -80^o^C until use.

### Immunoblotting assay

#### Protein extraction.

Tissues were weighed and kept on dry ice for sequential extraction into PBS and RIPA fractions. For the PBS extraction, tissues were manually homogenized with a hand-held homogenizer in PBS extraction buffer [Halt phosphatase inhibitor (#78420, Thermo Fisher Scientific) and Protease inhibitor cocktail III (#535140, EMD Millipore) in 1x PBS buffer] at 1:10 (w/v). Homogenates were centrifuged at 14,000 RPM for 30 minutes at 4^o^C, and the supernatant was removed as PBS-soluble fraction for storage at -80^o^C until use. The pellet was further extracted by the addition of RIPA extraction buffer [Halt phosphatase inhibitor, Protease inhibitor cocktail III, 50mM tris, 0.15M NaCl, 1% IGEPAL, 1mM EDTA, 1mM EGTA, 0.1% SDS and 0,5% sodium deoxylate in nanopure water, adjusted to pH 8.0] at 1:10 (w/v) and incubation on ice for 45 minutes. This was followed by sonication (Misonix XL-2000) at setting’5’ for 20 seconds on an ice-slurry and centrifugation at 14,000 RPM for 30 minutes at 4^o^C. The supernatant was removed as the RIPA-soluble fraction and stored at -80^o^C until use. Subsequently, the pellet was resuspended in 5 M guanidine (pH 8.0), sonicated for 20 seconds on ice, rotated for 1 hour at room temperature, and then, centrifuged at 14,000 RPM for 30 minutes at 4^o^C. Lastly, the supernatant containing the guanidine-soluble protein was stored in -80^o^C until analysis.

#### Western blot.

Sample protein concentrations were measured using bicinchoninic acid (BCA) assay (#23225, Thermo Fisher Scientific) followed by preparation of western blot samples by adding 4X NuPAGE LDS sample buffer (#NP0008, Thermo Fisher Scientific) with 4% β-Mercaptoethanol (#M6250, Millipore Sigma) to extracted proteins and heating at 95^o^C for 10 minutes. Samples containing equal amounts of protein were loaded into NuPAGE 4–12% midi Bis-tris gels (#WG1403BOX, Thermo Fisher Scientific) for running in 1x MOPS running buffer [50mM MOPS (#PHG0007, Millipore Sigma), 50mM Tris Base (#DST60040-10000, Dot Scientific), 1mM EDTA (#50-841-667, Teknova) and 0.1% SDS (#50-751-6948, Quality Scientific) in nanopure water] by using the Criterion Cell electrophoresis chamber (Bio-rad). Then, proteins were transferred to nitrocellulose membrane in 5x transfer buffer (Bio-rad) for 45 minutes at 1.1A and 25V using the Trans-Blot Turbo Transfer system (Bio-rad). The membrane was washed 3 times in Wash buffer (0.1% Tween-20 in 1x PBS) for 5 minutes per wash, and was blocked in SuperBlock blocking buffer (#37517, Thermo Fisher Scientific) at room temperature for 1 hour. Subsequently, the membrane was incubated with Primary antibody solution (Primary antibody and 10% SuperBlock blocking buffer in Wash buffer) overnight at 4^o^C. Next day, the membrane was washed 3 times in Wash buffer for 5 minutes per wash and was incubated with Secondary antibody solution (Secondary antibody and 10% SuperBlock blocking buffer in Wash buffer) at room temperature for 1 hour. The membrane was then washed 3 times in Wash buffer for 5 minutes at room temperature and developed with SuperSignal West Pico PLUS Chemiluminescent Substrate (#34580, Thermo Fisher Scientific) or SuperSignal West Femto Maximum Sensitivity Substrate (#34096, Thermo Fisher Scientific). Finally, membranes were imaged on a Bio-rad Chemidoc MP Imaging System for quantification analysis with the ImageLab Version 6.1 software.

#### Glycosylation.

PNGase F (#P0704L, New England Biolabs) was treated to deglycosylate ACE1 for assessment of the glycosylation status. The assay was performed in accordance with the manufacturer provided protocols. In brief, 1µl of 10X glycoprotein denaturing buffer was combined with 20 µg of protein and filled with water to a final 10µl reaction volume. This reaction was denatured by heating at 100^o^C for 10 minutes and subsequently placed on ice. The chilled reaction was centrifuged for 10 seconds and mixed with the following: 2µl of 10X GlycoBuffer 2, 2µl 10% NP-40, 6µl water, and 1µl PNGase F. Upon gently mixing the reaction, it was incubated at 37^o^C for 1 hour. Negative control was prepared identically to the protein samples but did not contain PNGase F in the reaction. Finally, samples were assessed by immunoblotting assay.

### Enzyme-linked immunosorbent assay (ELISA)

#### Angiotensin II ELISA.

Angiotensin II EIA Kit (#RAB0010, Sigma-Aldrich) was used to detect Angiotensin II (Ang II) peptide levels. The assay was performed in accordance with the manufacturer provided protocols. In brief, 100µl of diluted AngII antibody was added to each well of the precoated plate and was incubated overnight at 4^o^C with gentle shaking (1–2 cycles/second). Plates were then wash 4 times with 200µl of wash buffer per well. Next, 100µl of the protein samples, standards, positive control, or negative control was added to appropriate wells with a blank well. Each protein sample was prepared by combining 125µl of undiluted PBS-soluble protein sample (refer to the following methods section: ‘Immunoblotting assay’ – ‘Protein extraction’). with 125µl of biotinylated Angiotensin II Peptide at 40 pg/ml. Positive control was prepared by mixing 100µl of Angiotensin II Positive Control Sample, 100µl of biotinylated Angiotensin II Peptide at 40 pg/ml, and 4µl of 10-fold diluted biotinylated Angiotensin II Peptide. Negative control was prepared by combining 125µl of 1x PBS with 125µl of biotinylated Angiotensin II Peptide at 40 pg/ml. Standards were prepared at 1000, 100, 10, 1, 0.1, and 0 pg/ml by diluted EIA Angiotensin II Peptide standard with biotinylated Angiotensin II Peptide Working Stock at 20 pg/ml. Following this, the wells were covered with a film and incubated overnight at 4^o^C with gentle shaking (1–2 cycles/second). Next day, plates were washed 4 times with 200µl of wash buffer per well, followed by addition of 100µl of HRP-Streptavidin solution to each well for 45 minutes incubation with gentle shaking at room temperature. Plates were washed 4 times again with 200µl of wash buffer per well, and then, 100µl of TMB One-Step Substrate Reagent was added to each well for 30 minutes incubation at room temperature with gentle shaking. Lastly, 50µl of Stop Solution was added to each well and the plates were immediately measured for absorbance at 450nm.

#### Aβ40 ELISA.

Human Aβ40 ELISA Kit (#KHB3481, Invitrogen) was used to detect Aβ40 levels in the PBS, RIPA, and guanidine soluble fractions of the cortical brain homogenates. The assay was performed in accordance with the manufacturer provided protocols. In brief, 50µl of diluted Aβ40 peptide standard and samples were added to the wells that were precoated with the antibody. Standards were prepared at 500, 250, 125, 62.5, 31.25, 15.63, 7.81, and 0 pg/mL by diluting human Aβ40 peptide standard with Standard Diluent Buffer. Then, 50µl of Human Aβ40 Detection Antibody solution was added to each well, and the plate was covered and incubated for 3 hours at room temperature with gentle shaking at 100 RPM. The wells were then washed 4 times with 1X Wash Buffer. Subsequently, 100µl of Anti-Rabbit IgG HRP was added to each well and incubated for 30 minutes at room temperature. The wells were washed again 4 times with 1X Wash Buffer. Next, 100µl of Stabilized Chromogen was added to each well and incubated for 30 minutes at room temperature. Finally, 100µl of the Stop Solution was added to each well and measured for absorbance at 450nm.

#### Aβ42 ELISA.

Human Aβ42 ELISA Kit (#KHB3441, Invitrogen) were used to detect Aβ42 levels in the PBS, RIPA, and guanidine soluble fractions of the cortical brain homogenates. The assay was performed in accordance with the manufacturer provided protocols. In brief, 50µl of diluted Aβ42 peptide standard and samples were added to the wells that were precoated with the antibody. Standards were prepared at 1000, 500, 250, 125, 62.5, 31.25, 15.63, and 0 pg/mL by diluting human Aβ42 peptide standard with Standard Diluent Buffer. Then, 50µl of Human Aβ42 Detection Antibody solution was added to each well, and the plate was covered and incubated for 3 hours at room temperature with gentle shaking at 100 RPM. The wells were then washed 4 times with 1X Wash Buffer. Subsequently, 100µl of Anti-Rabbit IgG HRP was added to each well and incubated for 30 minutes at room temperature. The wells were washed again 4 times with 1X Wash Buffer. Next, 100µl of Stabilized Chromogen was added to each well and incubated for 30 minutes at room temperature. Finally, 100µl of the Stop Solution was added to each well and measured for absorbance at 450nm.

### Immunohistochemistry

#### Sectioning.

Hemibrains were frozen and sectioned at 30-microns thickness along the coronal or sagittal plane using a freezing-sliding microtome. These sections were serially harvested into a 12-well plate with cryoprotective solution (1x PBS, 30% sucrose, and 30% ethylene glycol) and preserved for long-term storage at -20^o^C.

#### Staining for immunofluorescence.

Brain sections from specific Bregma position were selected for each immunofluorescence analysis: NeuN, Aβ42, Iba1 and GFAP analysis (Bregma coordinates of approximately −1.70 to −3.52 mm) and brain volume analysis (Bregma coordinate of approximately −0.94 to −3.40 mm). Selected free-floating sections were placed into individual wells in a non-treated 24-well plate and were washed three times (5 minutes incubation per wash) in 1x tris-buffered saline (TBS) at room temperature with gentle shaking on an orbital shaker. Sections were then incubated in Glycine solution (16mM glycine and 0.25% Triton X-100 in 1x TBS) for 1 hour at room temperature with gentle shaking and were washed three times (5 minutes incubation per wash) in 1x TBS with gentle shaking. Next, sections were incubated in Blocking solution (0.25% Triton X-100, 5% donkey serum in 1x TBS) for either 2 hours at room temperature or overnight at 4^o^C with gentle shaking and were washed two times (10 minutes incubation per wash) in BSA solution (1% BSA and 0.25% Triton X-100 in 1x TBS). Sections were incubated in primary antibodies solution (primary antibodies, 1% BSA and 0.25% Triton X-100 in 1x TBS) overnight at 4^o^C with gentle shaking and were then washed three times (10 minutes incubation per wash) in BSA solution. Subsequently, sections were incubated in secondary antibodies solution (secondary antibody, 1% BSA and 0.25% Triton X-100 in 1x TBS) for 2 hours at room temperature with gentle shaking in the dark and were then washed three times (15 minutes incubation per wash) in 1x TBS. All sections were mounted onto slides (#MCOMM/W/90, StatLab) using ProLong Gold (#P36934, Thermo Fisher Scientific) with coverslips (#63791−01, Electron Microscopy Sciences) and dried overnight at room temperature.

#### Imaging and quantification for volumetric analysis.

Mounted anti-NeuN stained sections were imaged on the Ti2 wide-field microscope (Northwestern University Center for Advanced Microscopy and Nikon Imaging Centre). Sequential sections that were 360µm apart within Bregma coordinate of approximately −0.94 to −3.40 mm were selected for volumetric analysis. ImageJ software was used to trace and measure the area of interest (hippocampus, cortex, or cerebellum) on the section images. Finally, volume was calculated using the formula: volume = (sum of area) x 0.36 mm.

#### Imaging and quantification for NeuN, Aβ42, Iba1 and GFAP analysis.

Mounted anti-NeuN, anti-Aβ42, anti-Iba1 and anti-GFAP stained sections were imaged on the Ti2 wide-field microscope (Northwestern University Center for Advanced Microscopy and Nikon Imaging Centre). Nikon NIS-Elements Software (Northwestern University Nikon Imaging Centre) was used to set the image intensity and size thresholds and was also used to manually trace the area of interest (hippocampus, cortex, or cerebellum). A binary channel was created for each area of interest for running the analysis.

### Antibodies

#### Antibodies used for immunohistochemistry.

For immunohistochemistry, the following primary antibodies were used: beta Amyloid Recombinant Rabbit Monoclonal Antibody (H31L21, #700254, Thermo Fisher Scientific, RRID:AB_2532283), Anti-GFAP antibody (#ab4674, Abcam, RRID:AB_304558), Anti-Iba1 antibody (#ab107159, Abcam, RRID:AB_10972573), and Anti-NeuN Antibody (#ABN91, EMD Millipore, RRID:AB_11205592). Alexa Fluor-labeled secondary antibodies (Thermo Fisher Scientific; RRIDs specific to the fluorophore and host species are provided in the supplementary table).

**Antibodies used for immunoblotting assay:** Beta Amyloid Recombinant Rabbit Monoclonal Antibody (H31L21) (#700254, Thermo Fisher Scientific, RRID:AB_2533978), Recombinant Anti-Angiotensin Converting Enzyme 1 antibody [EPR22291-247] (#ab254222, Abcam, RRID:AB_3073965), ACE2 (E506J) XP Rabbit mAb (#92485, Cell Signaling Technology, no RRID available), β-Actin Rabbit Monoclonal Antibody (#926-42210, LI-COR, RRID:AB_1850027), and Goat Anti-Rabbit IgG Antibody (H+L), Peroxidase (#PI-1000-1, Vector Laboratories, RRID:AB_2916034).

### Statistical analysis

GraphPad Prism Version 9.4.1 (http://graphpad.com/scientific-software/prism/) was used for all statistical analysis. All data are presented as means ± SEM. Statistical significances were calculated by unpaired Student’s *t* test or ANOVA followed by Tukey’s post hoc test as detailed in the figure legends. *P* values less than 0.05 were considered significant: *P < 0.05, **P < 0.01, *** *P *< 0.001, and ****P < 0.0001.

## Results

### Genetic knockdown of ACE1 in 5XFAD mice reduces ACE1 levels

Our previous study reported accelerated cerebral Aβ accumulation in 5XFAD mice that express five familial AD mutations associated with amyloid precursor protein (APP) and presenilin1 (PS1) [4]. In these mice, amyloid deposition initiated at around 2-months of age and rapidly accumulated overtime [4]. Furthermore, these mice displayed characteristic AD phenotypes, such as spatial memory deficits, gliosis, and neurodegeneration [4]. In the current study, we sought to investigate the impact of ACE1 knockdown on amyloid burden by analyzing 6-month-old 5XFAD mice. This was mediated by crossing 5XFAD mice to neuronal ACE1 conditional knockout mice.

**Fig 1:**
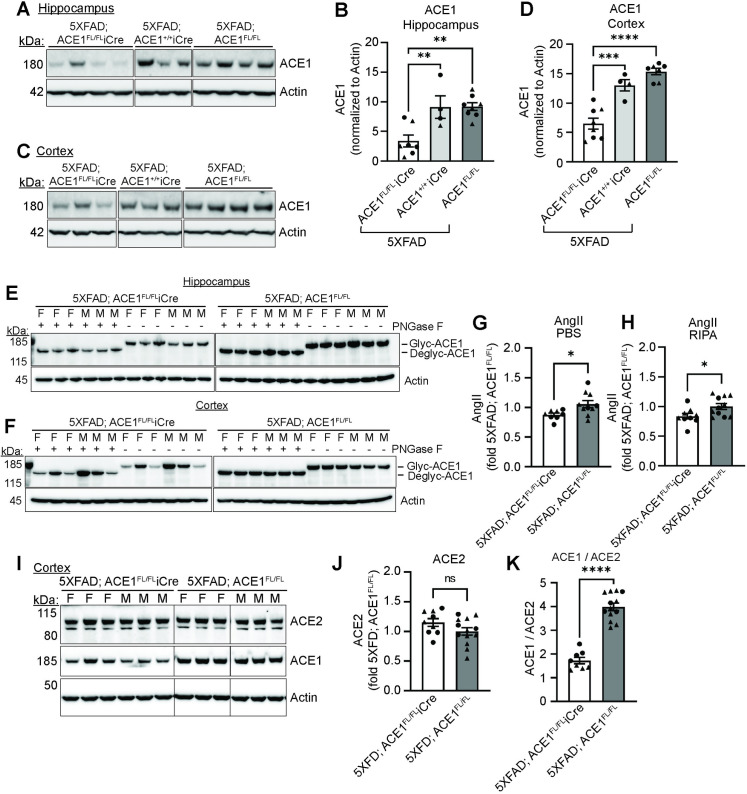
Genetic knockdown of ACE1 in 5XFAD mice reduces ACE1 protein levels. (A) Immunoblot of hippocampal homogenates from 6-months old 5XFAD; ACE1^FL/FL^iCre, 5XFAD; ACE1^+/+^iCre, and 5XFAD; ACE1^FL/FL^ mice probed for ACE1 and Actin. Uncropped blots in S1A Fig in S1 Raw Images. (B) Quantification of ACE1 in (A) normalized to Actin (5XFAD; ACE1^FL/FL^iCre, n = 7; 5XFAD; ACE1^+/+^iCre, n = 4; 5XFAD; ACE1^FL/FL^, n = 8). (C) Immunoblot of cortical homogenates from 6-months old 5XFAD; ACE1^FL/FL^iCre, 5XFAD; ACE1^+/+^iCre, and 5XFAD; ACE1^FL/FL^ mice probed for ACE1 and Actin. Uncropped blots in S1B Fig in S1 Raw Images. (D) Quantification of ACE1 in (C) normalized to Actin (5XFAD; ACE1^FL/FL^iCre, n = 8; 5XFAD; ACE1^+/+^iCre, n = 4; 5XFAD; ACE1^FL/FL^, n = 7). (E and F) RIPA-soluble fractions were treated with and without PNGase F to examine alterations in the glycosylation status of ACE1. Immunoblots of hippocampal **(E)** and cortical **(F)** homogenates from 6-months old 5XFAD; ACE1^FL/FL^iCre and 5XFAD; ACE1^FL/FL^ mice probed for ACE1 and Actin. Uncropped blots in S2 Fig in S1 Raw Images. (G and H) AngII levels were measured by ELISA from the cortical brain homogenates and normalized to µg of total protein in the samples. Quantification of AngII levels in the PBS **(G)** and RIPA **(H)** fraction of 5XFAD; ACE1^FL/FL^iCre or 5XFAD; ACE1^FL/FL^ mice at 6-months of age and expressed as fold of 5XFAD; ACE1^FL/FL^ (5XFAD; ACE1^FL/FL^iCre, n = 8-9; 5XFAD; ACE1^FL/FL^, n = 10-11). (I) Immunoblot of cortical homogenates from 6-months old 5XFAD; ACE1^FL/FL^iCre and 5XFAD; ACE1^FL/FL^ mice probed for ACE2, ACE1, and Actin. Uncropped blots in S3 Fig in S1 Raw Images. Quantification of ACE2 **(J)** and ACE1:ACE2 **(K)** normalized to Actin and expressed as fold of 5XFAD; ACE1^FL/FL^ (5XFAD; ACE1^FL/FL^iCre, n = 9; 5XFAD; ACE1^FL/FL^, n = 13). One-way ANOVA with Tukey’s multiple comparisons post hoc test in (B) and (D). Unpaired t test in (G), (H), (J), and (K). Circles represent data for female and triangles for male mice. *P < 0.05, **P < 0.01, ***P < 0.001, ****P < 0.0001. Abbreviations (F = females; M = males).

First, to examine 5XFAD mice with ACE1 genetic knockdown, we crossed 5XFAD mice with ACE1^FL/FL^iCre mice to generate 5XFAD; ACE1^FL/FL^iCre mice. Our recent study on ACE1^FL/FL^iCre mice, whereby neuronal ACE1 was knocked out in excitatory forebrain neurons, showed a reduction of total ACE1 reaching 65% and 55% reduction in ACE1 levels at the hippocampus and the cortex, respectively, compared to that of controls [13]. To confirm ACE1 genetic knockdown in 5XFAD; ACE1^FL/FL^iCre mice, we analyzed hippocampal and cortical homogenates of 5XFAD; ACE1^+/+^iCre mice and 5XFAD; ACE1^FL/FL^ control mice. Compared to controls, 5XFAD; ACE1^FL/FL^iCre mice showed around 63% and 50–58% reduction in ACE1 levels at the hippocampus and cortex respectively, indicating partial knockdown of ACE1 in the excitatory forebrain neurons of 5XFAD; ACE1^FL/FL^iCre mice (Fig 1A–D). Together, our initial analysis confirmed genetic knockdown of ACE1 in 5XFAD; ACE1^FL/FL^iCre mice.

ACE1 is a transmembrane glycoprotein, and the glycosylation status of ACE1 impacts its stability and catalytic activity, such as angiotensin-converting-activity and Aβ cleavage [23]. To assess the glycosylation status of ACE1, we treated 5XFAD; ACE1^FL/FL^iCre and 5XFAD; ACE1^FL/FL^ mice with PNGase F (Fig 1E,F). We observed no alterations in the glycosylation pattern between these mice in both the hippocampus and the cortex, suggesting that ACE1 is glycosylated and should be catalytically active in these mice (Fig 1E,F). Subsequently, we measured the levels of AngII, a downstream catalytic product of ACE1 in the RAS, and detected a reduction in AngII levels of around 17% in both the PBS and RIPA homogenates in 5XFAD; ACE1^FL/FL^iCre compared to control mice (Fig 1G,H). This suggested that ACE1 is catalytically active and further confirmed successful knockdown of ACE1 in our 5XFAD; ACE1^FL/FL^iCre mice. Additionally, we assessed the levels of ACE2, which is another component of the RAS, to determine whether its levels are counterbalanced with the reduction in ACE1 levels (Fig 1I–K). Despite the decreased ACE1 levels, the ACE2 levels were unaltered in the cortical homogenates of 5XFAD; ACE1^FL/FL^iCre when compared to that of 5XFAD; ACE1^FL/FL^ mice (Fig 1I–K). Altogether, these results suggest that, in these mice, ACE1 is functional and further implies that our downstream analyses are dependent on the effect of ACE1, but not ACE2.

In our previous study, we reported that ACE1 KI mice have elevated ACE1 and significantly reduced hippocampal volume at 14-months of age, but not at 8-months of age [10]. This age- dependent hippocampal volume loss was rescued by treatment with captopril, an ACE1 inhibitor (ACEi) [10]. To determine the neuronal and physiological effects of neuronal ACE1 knockdown in 5XFAD mice, we measured the brain and body weights of 5XFAD; ACE1^FL/FL^ mice compared to 5XFAD; ACE1^FL/FL^iCre. Compared to 5XFAD; ACE1^FL/FL^ mice, 5XFAD; ACE1^FL/FL^iCre mice showed no significant changes in both brain and body weights (S6 Fig). Thus, these experiments show that neuronal ACE1 knockdown does not cause alterations in the brain and body weight in 5XFAD mice.

### Genetic ACE1 knockdown does not affect cerebral amyloid plaque load in the cortex and hippocampus of 5XFAD mice

**Fig 2:**
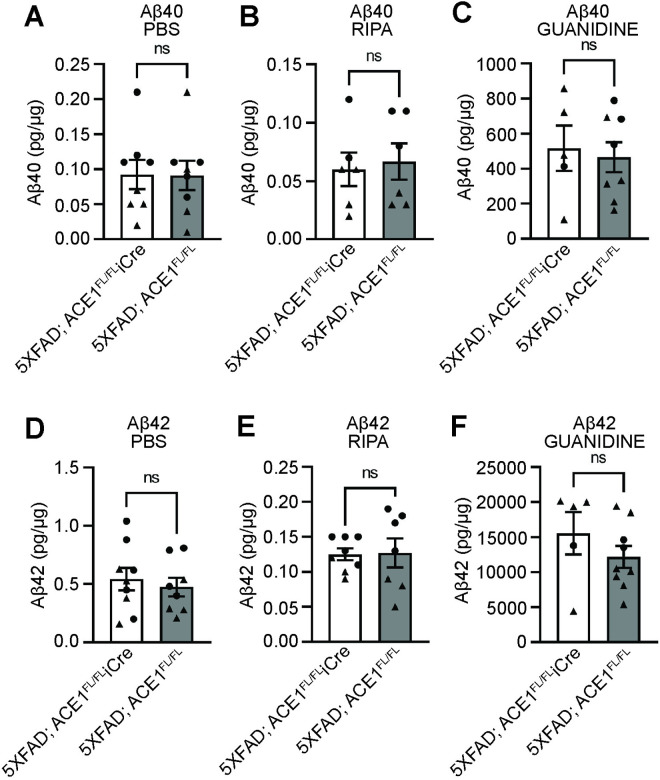
Genetic knockdown of ACE1 in 5XFAD mice does not affect Aβ40 and Aβ42 levels in the cortex. (A to C) Quantification of Aβ40 levels as measured by ELISA in the PBS (A), RIPA (B), and Guanidine **(C)** fractions of the cortical brain homogenates of 5XFAD; ACE1^FL/FL^iCre or 5XFAD; ACE1^FL/FL^ mice at 6-months of age. Values were normalized to µg of total protein in the sample. (5XFAD; ACE1^FL/FL^iCre, n = 6-8; 5XFAD; ACE1^FL/FL^, n = 6-8). (D to F) Quantification of Aβ42 levels as measured by ELISA in the PBS (D), RIPA (E), and Guanidine **(F)** fractions of the cortical brain homogenates of 5XFAD; ACE1^FL/FL^iCre or 5XFAD; ACE1^FL/FL^ mice at 6-months of age. Values were normalized to µg of total protein in the sample. (5XFAD; ACE1^FL/FL^iCre, n = 5-9; 5XFAD; ACE1^FL/FL^, n = 7-8). Unpaired *t* test in (A) to (F). Circles represent data for female and triangles for male mice. *P < 0.05, **P < 0.01, ***P < 0.001, ****P < 0.0001.

To evaluate whether ACE1 impacts amyloid accumulation in vivo, we performed quantitative analyses of Aβ40 and Aβ42 levels using ELISA across PBS, RIPA, and guanidine fractions from cortical tissue. PBS, RIPA, and guanidine fractions were used to sequentially extract Aβ species of differing solubility from brain tissue. This sequential extraction allowed us to perform a comprehensive analysis of Aβ in its various biochemical states. Here, we measured Aβ40 and Aβ42 levels in the PBS, RIPA, and guanidine fractions (Fig 2). Both Aβ40 and Aβ42 levels in the PBS and RIPA soluble cortical homogenates were unaltered between 5XFAD; ACE1^FL/FL^iCre and 5XFAD; ACE1^FL/FL^ mice, suggesting that neuronal ACE1 knockdown does not impact soluble oligomeric amyloid beta (Fig 2A,B,D,E). Further assessment of the Aβ40 and Aβ42 levels in the guanidine soluble fraction also showed no significant difference in 5XFAD; ACE1^FL/FL^iCre compared to control mice (Fig 2C,F). Therefore, these results suggest that neuronal ACE1 partial knockdown does not affect amyloid plaque accumulation in the cortex of 5XFAD mice at 6-months of age.

**Fig 3:**
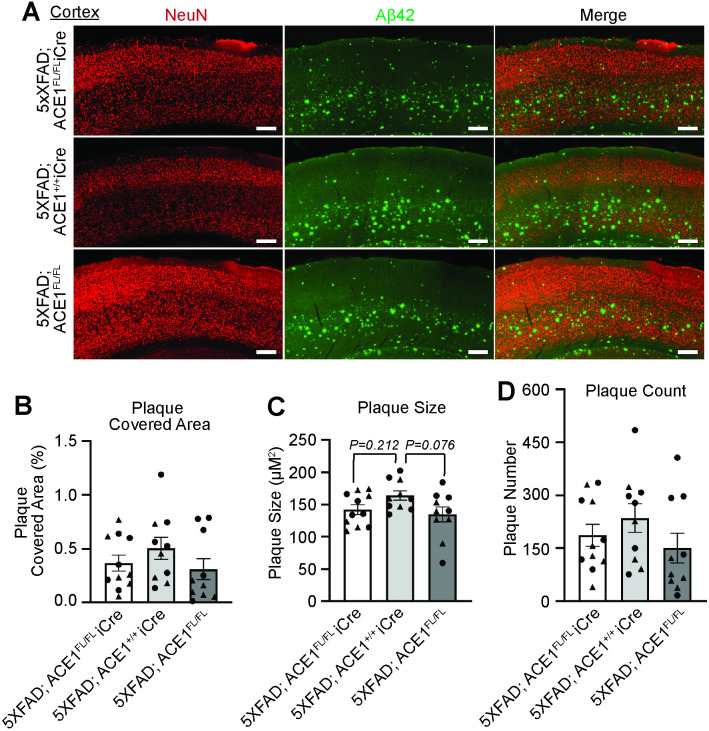
ACE1 knockdown does not affect cortical amyloid plaque load as examined by Aβ42 immunostaining in 5XFAD mice. (A) Representative images showing layer 5 cortex of coronal sections from 6-months-old 5XFAD; ACE1^FL/FL^iCre, 5XFAD; ACE1^+/+^iCre, and 5XFAD; ACE1^FL/FL^ mice immunostained for NeuN (red) and Aβ42 (green) imaged by confocal immunofluorescence microscopy. Scale bar, 200μm. Quantification of plaque covered area (B), plaque size (C), and plaque count (D) in (A). (5XFAD; ACE1^FL/FL^iCre, n = 11; 5XFAD; ACE1^+/+^iCre, n = 10; 5XFAD; ACE1^FL/FL^, n = 10). One-way ANOVA with Tukey’s multiple comparisons post hoc test was performed in (B to D). Circles represent data for female and triangles for male mice. *P < 0.05, **P < 0.01, ***P < 0.001, ****P < 0.0001.

Although multiple *in vitro* studies support ACE1’s role in Aβ degradation, clinical and *in vivo* studies report conflicting results [18–27,29–33,38–40,43]. To interrogate whether a reduction in neuronal ACE1 protein levels affects Aβ burden *in vivo*, we examined amyloid plaque load as measured by Aβ42 levels through immunofluorescence microscopy in both the cortex and the hippocampus. Since previous studies on 5XFAD mice reported that amyloid plaques first appear in the deep layers of the cortex, we first examined the cortical brain regions for potential changes in plaque accumulation at 6-months of age [4]. We found no significant differences in the plaque covered area, size and count between 5XFAD; ACE1^FL/FL^iCre, 5XFAD; ACE1^+/+^iCre, and 5XFAD; ACE1^FL/FL^ mice in the cortex (Figs 3 and S4). These results suggest that ACE1 partial knockdown does not affect deposited, insoluble amyloid beta.

**Fig 4:**
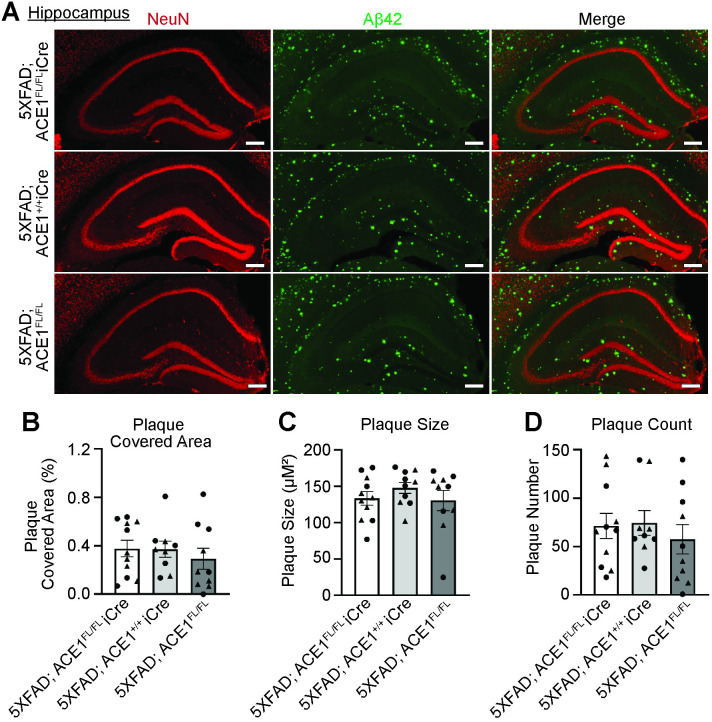
ACE1 genetic knockdown does not affect hippocampal amyloid plaque load as examined by Aβ42 immunostaining in 5XFAD mice. (A) Representative images showing the hippocampus of coronal sections from 6-months-old 5XFAD; ACE1^FL/FL^iCre, 5XFAD; ACE1^+/+^iCre, and 5XFAD; ACE1^FL/FL^ mice immunostained for NeuN (red) and Aβ42 (green) imaged by confocal immunofluorescence microscopy. Scale bar, 200μm. Quantification of plaque covered area (B), plaque size (C), and plaque count **(D)** in (A). (5XFAD; ACE1^FL/FL^iCre, n = 11; 5XFAD; ACE1^+/+^iCre, n = 9-10; 5XFAD; ACE1^FL/FL^, n = 10). One-way ANOVA with Tukey’s multiple comparisons post hoc test was performed in (B to D). Circles represent data for female and triangles for male mice. *P < 0.05, **P < 0.01, ***P < 0.001, ****P < 0.0001.

In addition to the cortical brain regions, we further examined the hippocampal brain regions, which is another vulnerable brain region in AD. Our recent study on mice with ACE1 conditional knockout in the excitatory forebrain neurons revealed hippocampal dependent behavioral deficits and showed RAS dysregulation and cerebrovascular defects selectively in the hippocampal brain regions [13]. Moreover, our ACE1 KI mice displayed hippocampal neurodegeneration which was rescued by ACEi administration [10]. These studies suggest an impact of ACE1 selectively on the hippocampus in the AD brain and prompted us to investigate whether ACE1 affects hippocampal amyloid plaque accumulation. Our analysis of plaque covered area, size and count revealed no significant differences between 5XFAD; ACE1^FL/FL^iCre, 5XFAD; ACE1^+/+^iCre and 5XFAD; ACE1^FL/FL^ mice in the hippocampus (Figs 4 and S5). These analyses suggest that neuronal ACE1 does not appear to affect hippocampal amyloid plaque accumulation.

### ACE1 partial loss of function does not affect neuroinflammation in the hippocampus and cortex of 5XFAD mice

Multiple studies report neuroinflammation in AD transgenic mouse models, including 5XFAD mice wherein astrocytes were closely associated with amyloid deposits in the brain [4]. ACE1 KI mice also showed neuroinflammation, which was accelerated in 5XFAD background but attenuated with ACEi administration that was caused by elevated baseline levels of AngII [10]. We next explored whether neuronal ACE1 knockdown in 5XFAD mice impacts neuroinflammation in the brain. To investigate this, we examined glial fibrillary acidic protein (GFAP) and ionized calcium binding adaptor molecule 1 (Iba1) immunoreactivity as a measure for astrocytic and microglial activation.

**Fig 5:**
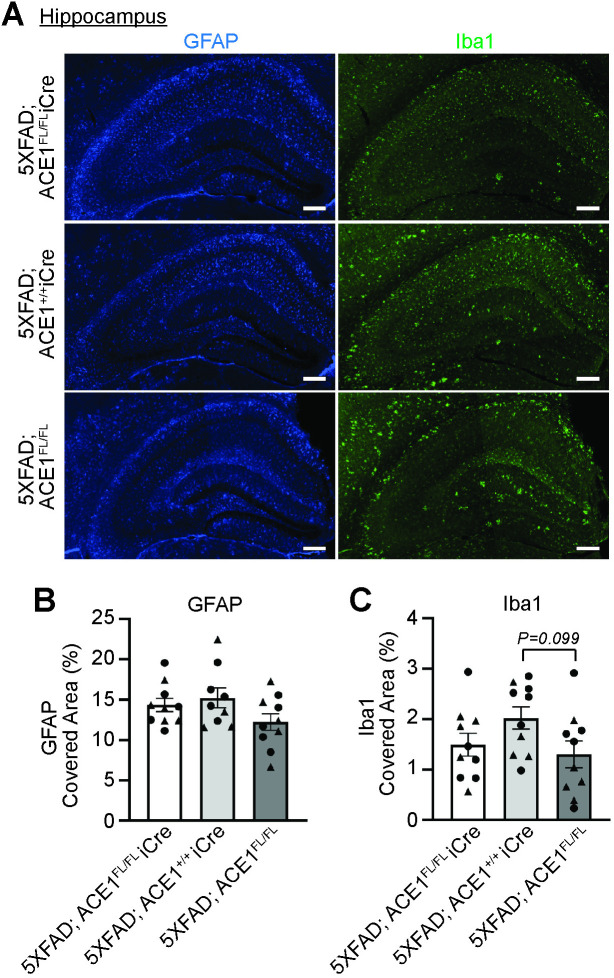
Hippocampal neuroinflammation is unaffected in 5XFAD mice with ACE1 genetic knockdown. (A) Representative images showing the hippocampus of coronal sections from 6-months-old 5XFAD; ACE1^FL/FL^iCre, 5XFAD; ACE1^+/+^iCre, and 5XFAD; ACE1^FL/FL^ mice immunostained for GFAP (blue) and Iba1 (green) imaged by confocal immunofluorescence microscopy. Scale bar, 200μm. Quantification of GFAP covered area **(B)** and Iba1 covered area **(C)** in (A). (5XFAD; ACE1^FL/FL^iCre, n = 10; 5XFAD; ACE1^+/+^iCre, n = 9-10; 5XFAD; ACE1^FL/FL^, n = 10). One-way ANOVA with Tukey’s multiple comparisons post hoc test was performed in (B) and (C). Circles represent data for female and triangles for male mice. *P < 0.05, **P < 0.01, ***P < 0.001, ****P < 0.0001.

We first examined the hippocampal brain regions where astrogliosis and microgliosis have been observed in both 5XFAD and ACE1 KI mice based on our previous studies [4,10]. Immunofluorescence microscopy analysis showed no statistically significant differences in the GFAP and Iba1 covered area in the hippocampus between 5XFAD; ACE1^FL/FL^iCre, 5XFAD; ACE1^+/+^iCre and 5XFAD; ACE1^FL/FL^ mice (Fig 5). Thus, neuronal ACE1 partial loss of function in the hippocampus does not affect neuroinflammation in 5XFAD mice.

**Fig 6:**
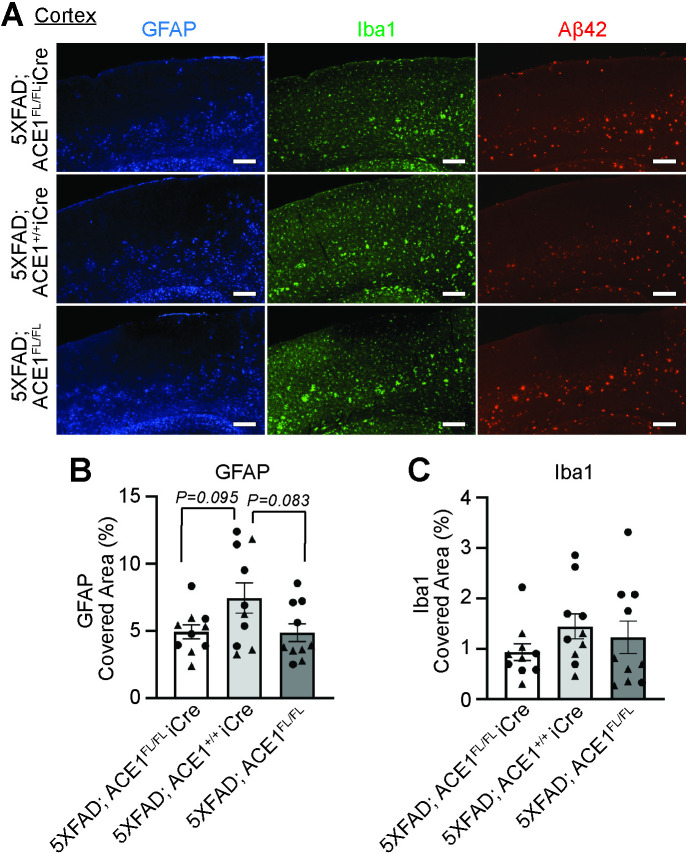
Cortical neuroinflammation is unaffected in 5XFAD mice with ACE1 genetic knockdown. (A) Representative images showing layer 5 cortex of coronal sections from 6-months-old 5XFAD; ACE1^FL/FL^iCre, 5XFAD; ACE1^+/+^iCre, and 5XFAD; ACE1^FL/FL^ mice immunostained for GFAP (blue), Iba1 (green) and Aβ42 (red) imaged by confocal immunofluorescence microscopy. Scale bar, 200μm. Quantification of GFAP covered area **(B)** and Iba1 covered area **(C)** in (A). (5XFAD; ACE1^FL/FL^iCre, n = 10-11; 5XFAD; ACE1^+/+^iCre, n = 10; 5XFAD; ACE1^FL/FL^, n = 10). One-way ANOVA with Tukey’s multiple comparisons post hoc test was performed in (B) and (C). Circles represent data for female and triangles for male mice. *P < 0.05, **P < 0.01, ***P < 0.001, ****P < 0.0001.

Robust gliosis has been also observed in the cortical brain regions in both 5XFAD in response to amyloidosis and in mice with ACE1 KI mutations that have increased AngII level above baseline [4,10]. Here, our analysis did not detect significant changes in cortical astrogliosis and microgliosis between 5XFAD; ACE1^FL/FL^iCre, 5XFAD; ACE1^+/+^iCre and 5XFAD; ACE1^FL/FL^ mice as measured by GFAP and Iba1 coverage (Fig 6). Together, in 5XFAD mice, neuroinflammation is unaffected with neuronal ACE1 partial knockdown in the cortical brain regions.

## Discussion

In this study, we interrogated the discrepancies regarding the role of ACE1 in Aβ accumulation. We examined this *in vivo* in a novel 5XFAD mouse model where neuronal ACE1 was partially knocked down. Our results show that, in the 5XFAD mouse model, neuronal ACE1 knockdown does not alter Aβ levels, suggesting that neuronal ACE1 does not appear to play a significant role in Aβ degradation *in vivo*.

The major finding in this study is that neuronal ACE1 partial knockdown in the transgenic AD brain does not cause a significant alteration in amyloid burden. This conflicts with most of the existing *in vitro* studies reporting ACE1’s role in Aβ degradation [19–25]. In contrast, our results are in line with several *in vivo* and clinical studies suggesting that ACE1 does not affect Aβ levels in the brain [24,38–40,42–45]. Several studies on AD transgenic mice revealed no significant differences in cerebral Aβ levels with ACE1 inhibitors; in particular, CD-1 mice injected with enalaprilat did not alter Aβ40 and Aβ42 levels [38–41]. In addition, multiple lines of evidence from clinical studies report no significant differences in AD incidence with ACE1 inhibitor as a class [36,43,44]. Furthermore, investigation of AT1R-null AD transgenic mice showed reduced amyloid pathology, but ACE1 levels remained unaltered [42]. Therefore, our findings substantiate some clinical and *in vivo* studies but not *in vitro* reports.

Our physiological and functional studies demonstrate that neuronal ACE1 partial knockdown in 5XFAD mice does not cause AD neuropathology, which was unexpected. Based on the canonical RAS pathway, ACE1 knockout should reduce AngII level and subsequently reduce AT1R activation levels. Multiple studies show that administration of AT1R blockers (ARBs), such as losartan and candesartan, rescues neuroinflammation, oxidative stress, and improves cognitive impairment; thus, AT1R activation may accelerate neurodegeneration [46–48]. Furthermore, AT1R activation is also known to cause vasoconstriction and reduce cerebral blood flow, and the resulting cerebral hypoxia may impact the production of Aβ through regulating enzymes such as BACE1 [49,50]. Indeed, postmortem AD patient brain analysis revealed a link between elevated AT1R levels with Aβ levels [51,52]. Given these roles of AT1R activation, we expected to see rescue in AD neuropathology, such as neuroinflammation and amyloid plaque accumulation, in 5XFAD mice through AT1R blockage mediated by ACE1 inhibition [18,53]. Our expectation was also based on previous studies where BBB-penetrating ACE1 inhibitor, perindopril, has been shown to attenuate neuroinflammation, reduce Aβ levels and increase cerebral blood flow [54–56]. The cerebral RAS is yet to be fully understood, and we hypothesize that our unexpected results may be due to compensatory mechanisms. To explain, compared to control mice, 5XFAD; ACE1^FL/FL^iCre mice showed over 50% reduction in ACE1 protein levels in the hippocampus and the cortex. However, although significant, the corresponding reduction in AngII levels was only 17%, and this is likely due to the compensated AngII levels. For example, in the noncanonical RAS pathway, cathepsin G or tonin can directly produce AngII from angiotensinogen (AGT) [12].In addition, AngII is also known to bind to AT2R, which counteracts the effects of AT1R and counterbalances downstream phenotypes such as neuroinflammation. Thus, despite neuronal ACE1 knockdown, this unaltered AT1R activation through compensated AngII levels may have led to no rescue of neuroinflammation and brain atrophy as observed in our data.

There are several limitations to our study. First, the effect of neuronal ACE1 knockdown in 5XFAD mice was assessed only at 6-months of age and not examined at other timepoints in our study. Compared to other transgenic AD mice, 5XFAD mice display accelerated Aβ deposition starting from 2-months of age. Therefore, we assessed our mice at 6-months of age when there is significant Aβ deposition; however, it is possible that we may have seen a difference at earlier or later timepoints. Second, our study relies on Aβ40 and Aβ42 levels as a measure for amyloid burden. Since amyloid plaque is not restricted to Aβ40 and Aβ42 alone, it may not have been a comprehensive assessment as evidenced in previous studies. Other Aβ measurements such as thioflavin S and western blotting may be utilized for future studies. In addition, recognize that direct assessment of Aβ catabolism would require analysis of specific cleavage products generated by ACE1, which was not performed in this study. Most importantly, we characterized 5XFAD mice with ACE1 partial knockdown specifically in the excitatory forebrain neurons, but not in other cell types. Therefore, ACE1 knockout or inhibition at a wider range of cell types, such as endothelial cells and/or glial cells, may affect brain volumes and amyloid burden.

## Conclusions

In conclusion, neuronal ACE1 does not appear to affect plaque accumulation in 5XFAD transgenic AD mouse model. Therefore, the role of neuronal ACE1 in AD pathogenesis may be through alternative pathways that remain to be explored in future studies.

## Supporting information

S1 Raw Images S1-S3 Figs. Compiled original uncropped and unadjusted raw western blot images. (PDF)

S4 Fig Cortical plaque covered area, size and count are unaltered in both 5XFAD males and females with ACE1 genetic knockdown or enalapril. (A to C) Imaging analysis of coronal sections from the cortex of 6-months-old 5XFAD; ACE1^FL/FL^iCre, 5XFAD; ACE1^+/+^iCre, and 5XFAD; ACE1^FL/FL^ mice. Quantification of plaque covered area (A), plaque size (B), and plaque count (C) in (Fig. 3) through independent analysis of females (left) and males (right). Females (5XFAD; ACE1^FL/FL^iCre, n = 5; 5XFAD; ACE1^+/+^iCre, n = 6; 5XFAD; ACE1^FL/FL^, n = 5). Males (5XFAD; ACE1^FL/FL^iCre, n = 6; 5XFAD; ACE1^+/+^iCre, n = 4; 5XFAD; ACE1^FL/FL^, n = 5). One-way ANOVA with Tukey’s multiple comparisons post hoc test was performed in (A to C). Circles represent data for female and triangles for male mice. *P < 0.05, **P < 0.01, ***P < 0.001, ****P < 0.0001.
(TIF)

S5 Fig Hippocampal plaque covered area, size and count are unchanged in both 5XFAD males and females with ACE1 genetic knockdown. (A to C) Imaging analysis of coronal sections from the hippocampus of 6-months-old 5XFAD; ACE1^FL/FL^iCre, 5XFAD; ACE1^+/+^iCre, and 5XFAD; ACE1^FL/FL^ mice. Quantification of plaque covered area (A), plaque size (B), and plaque count (C) in (Fig. 4) through independent analysis of females (left) and males (right). Females (5XFAD; ACE1^FL/FL^iCre, n = 5; 5XFAD; ACE1^+/+^iCre, n = 5–6; 5XFAD; ACE1^FL/FL^, n = 5). Males (5XFAD; ACE1^FL/FL^iCre, n = 6; 5XFAD; ACE1^+/+^iCre, n = 4; 5XFAD; ACE1^FL/FL^, n = 5). One-way ANOVA with Tukey’s multiple comparisons post hoc test was performed in (A to C). Circles represent data for female and triangles for male mice. *P < 0.05, **P < 0.01, ***P < 0.001, ****P < 0.0001.
(TIF)

S6 Fig ACE1 deficit does not significantly alter the brain and body weight of 5XFAD mice. (A) Quantification of the brain weights (mg) from 6-months old 5XFAD; ACE1^FL/FL^iCre and 5XFAD; ACE1^FL/FL^ mice. (5XFAD; ACE1^FL/FL^iCre, n = 18; 5XFAD; ACE1^FL/FL^, n = 19). (B) Quantification of the body weights (g) from 6-months old 5XFAD; ACE1^FL/FL^iCre and 5XFAD; ACE1^FL/FL^ mice (5XFAD; ACE1^FL/FL^iCre, n = 18; 5XFAD; ACE1^FL/FL^, n = 19). Unpaired *t* test in (A to B). Circles represent data for female and triangles for male mice. *P < 0.05, **P < 0.01, ***P < 0.001, ****P < 0.0001.
(TIF)

S7 Fig Raw values behind the means, standard deviations and other measures reported used to build graphs. (XLSX)

## Abbreviations:

Aβ42: Amyloid Beta 42 peptide
ACE1: Angiotensin Converting Enzyme 1
ACEi: ACE1 inhibitor
AD: Alzheimer’s Disease
AGT: Angiotensinogen
AngI: Angiotensin I
AngII: Angiotensin II
APP: Amyloid Precursor Protein
AT1R: AngII Receptor Type 1
AT2R: AngII Receptor Type 2
BBB: Blood Brain Barrier
CER: Cerebellum
CNS: Central Nervous System
CTX: Cortex
FAD: Familial Alzheimer’s Disease
HPC: Hippocampus
PS1: Presenilin1
RAS: Renin Angiotensin System
