# Supplementary material for: ACE1 does not influence cerebral Aβ degradation or amyloid plaque accumulation in 5XFAD mice
Source: PLoS One. 2025 Sep 15;20(9):e0330193. doi: 10.1371/journal.pone.0330193 (PMC12435669; doi:10.1371/journal.pone.0330193)
Supplement: S1 Raw Images — (PDF) [file pone.0330193.s001.pdf]

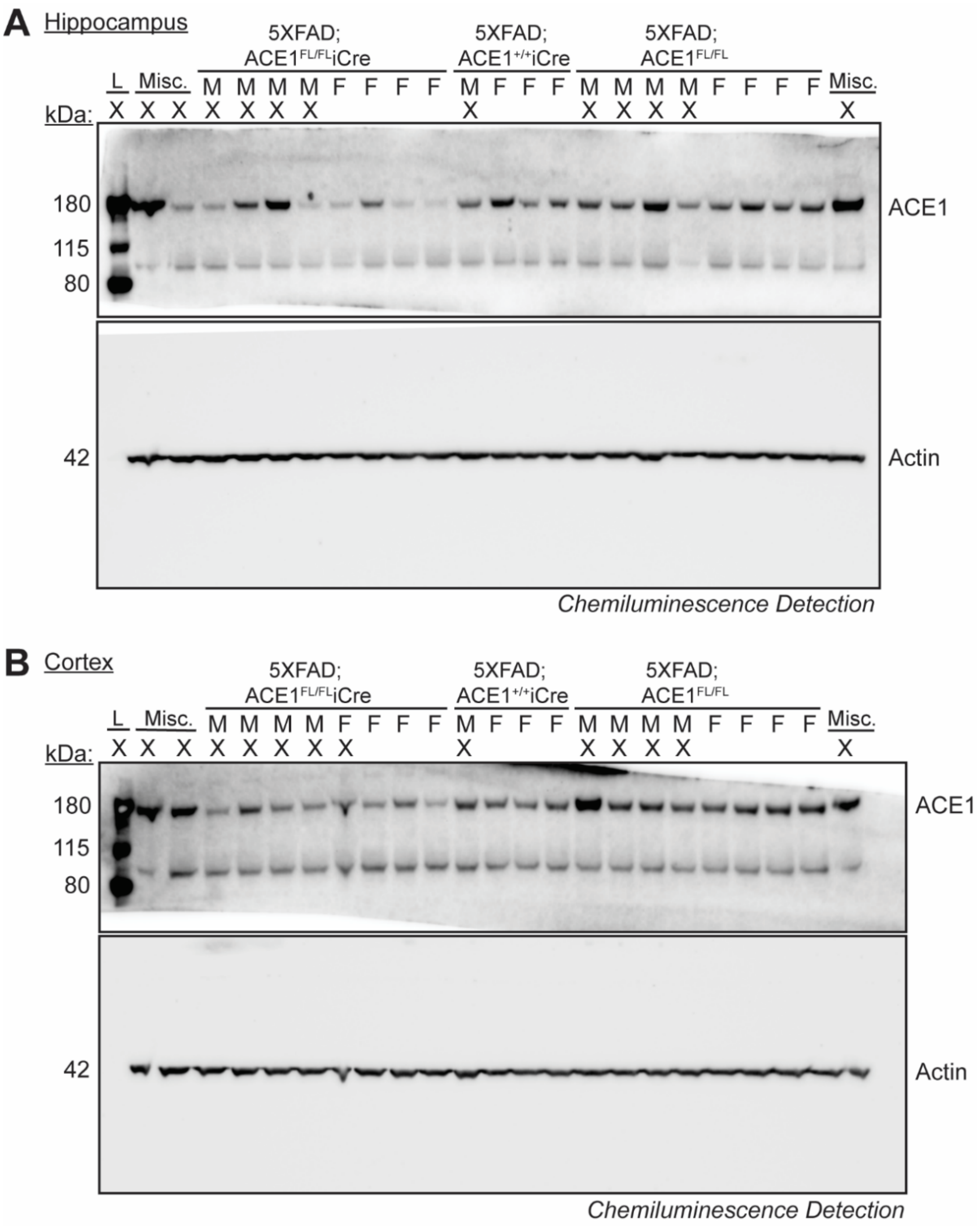

S1 Fig. Original blots of ACE1 shown in Main Figure 1.

(A) Original (uncropped) immunoblots (of Fig. 1A) showing hippocampal homogenates from 6-months old 5XFAD; ACE1$^{FL/FL}$iCre, 5XFAD; ACE1$^{+/+}$iCre, and 5XFAD; ACE1$^{FL/FL}$ mice probed for ACE1 and Actin. (5XFAD; ACE1$^{FL/FL}$iCre, n=7; 5XFAD; ACE1$^{+/+}$iCre,

n=4; 5XFAD; ACE1$^{FL/FL}$, n=8). **(B)** Original immunoblots (of Fig. 1C) showing cortical homogenates from 6-months old 5XFAD; ACE1$^{FL/FL}$iCre, 5XFAD; ACE1$^{+/+}$iCre, and 5XFAD; ACE1$^{FL/FL}$ mice probed for ACE1 and Actin. (5XFAD; ACE1$^{FL/FL}$iCre, n=8; 5XFAD; ACE1$^{+/+}$iCre, n=4; 5XFAD; ACE1$^{FL/FL}$, n=7). Images were captured by chemiluminescence detection. Abbreviations (L = protein ladder; Misc. = miscellaneous sample; F = females; M = males; X = lane not included in the final figure).

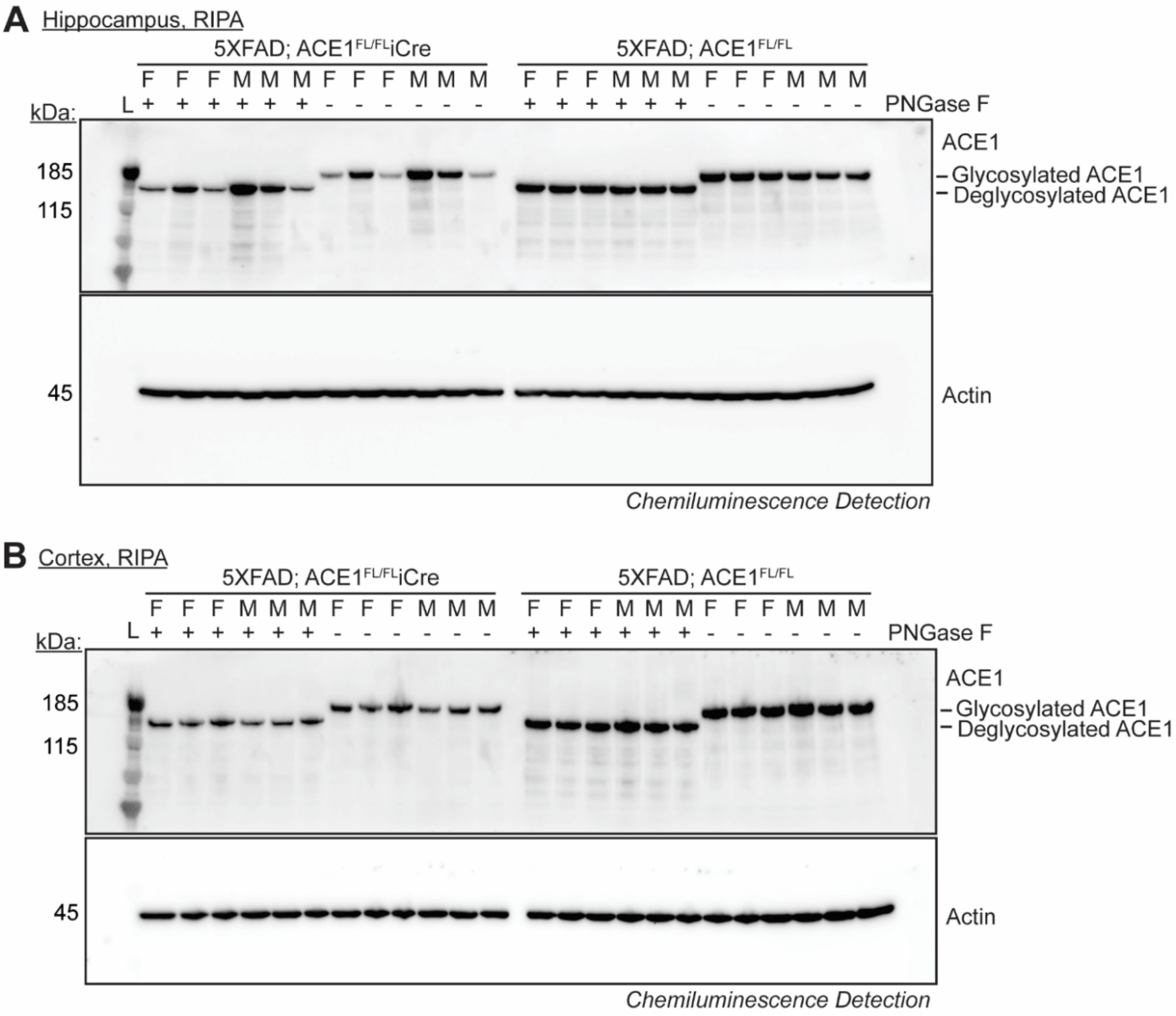

S2 Fig. Original blots of ACE1 glycosylation status shown in Main Figure 1.

(A) Original (uncropped) immunoblots (of Fig. 1E) showing hippocampal homogenates from 6-months old 5XFAD; ACE1$^{FL/FL}$iCre and 5XFAD; ACE1$^{FL/FL}$ mice probed for ACE1 and Actin. (5XFAD; ACE1$^{FL/FL}$iCre, n=6; 5XFAD; ACE1$^{FL/FL}$, n=6). **(B)** Original (uncropped) immunoblots (of Fig. 1F) showing cortical homogenates from 6-months old 5XFAD; ACE1$^{FL/FL}$iCre and 5XFAD; ACE1$^{FL/FL}$ mice probed for ACE1 and Actin. (5XFAD; ACE1$^{FL/FL}$iCre, n=6; 5XFAD; ACE1$^{FL/FL}$, n=6). Images were captured by chemiluminescence detection. Abbreviations (L = protein ladder; F = females; M = males).

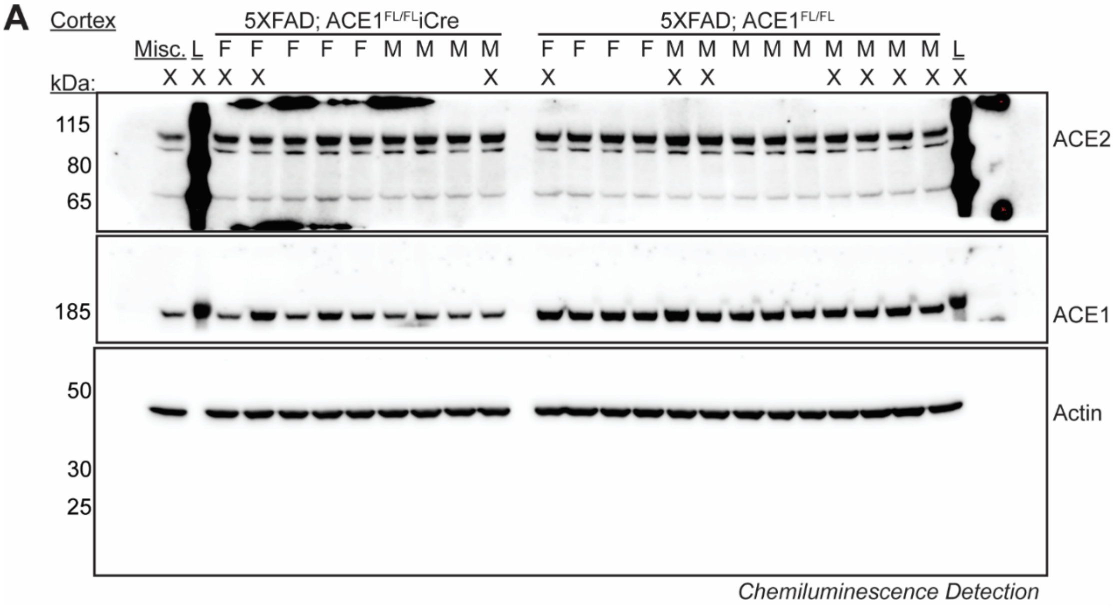

S3 Fig. Original blots of ACE2 shown in Main Figure 1.

Original (uncropped) immunoblots (of Fig. 1I) showing cortical homogenates from 6-months old 5XFAD; ACE1$^{FL/FL}$iCre and 5XFAD; ACE1$^{FL/FL}$ mice probed for ACE2, ACE1 and Actin. (5XFAD; ACE1$^{FL/FL}$iCre, n=9; 5XFAD; ACE1$^{FL/FL}$, n=13). Images were captured by chemiluminescence detection. Abbreviations (L = protein ladder; Misc. = miscellaneous; F = females; M = males; X = lane not included in the final figure).
