# Supplementary figures and images for: ACE1 does not influence cerebral Aβ degradation or amyloid plaque accumulation in 5XFAD mice
Source: PLoS One. 2025 Sep 15;20(9):e0330193. doi: 10.1371/journal.pone.0330193 (PMC12435669; doi:10.1371/journal.pone.0330193)

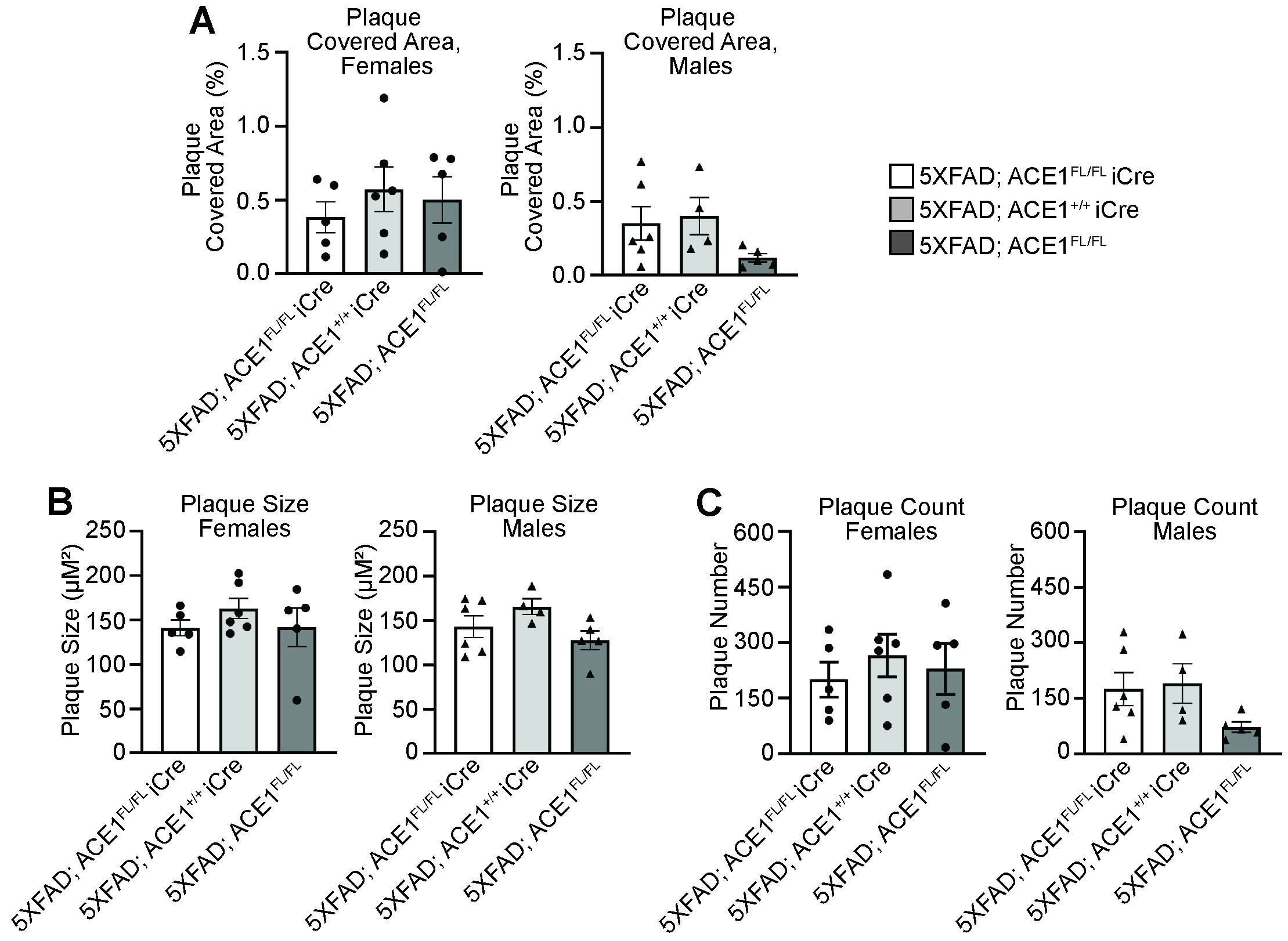

Supplement: S4 Fig — (A to C) Imaging analysis of coronal sections from the cortex of 6-months-old 5XFAD; ACE1FL/FLiCre, 5XFAD; ACE1+/+iCre, and 5XFAD; ACE1FL/FL mice. Quantification of plaque covered area (A), plaque size (B), and plaque count (C) in (Fig. 3) through independent analysis of females (left) and males (right). Females (5XFAD; ACE1FL/FLiCre, n = 5; 5XFAD; ACE1+/+iCre, n = 6; 5XFAD; ACE1FL/FL, n = 5). Males (5XFAD; ACE1FL/FLiCre, n = 6; 5XFAD; ACE1+/+iCre, n = 4; 5XFAD; ACE1FL/FL, n = 5). One-way ANOVA with Tukey’s multiple comparisons post hoc test was performed in (A to C). Circles represent data for female and triangles for male mice. *P < 0.05, **P < 0.01, ***P < 0.001, ****P < 0.0001. (TIF) [file pone.0330193.s002.tif]

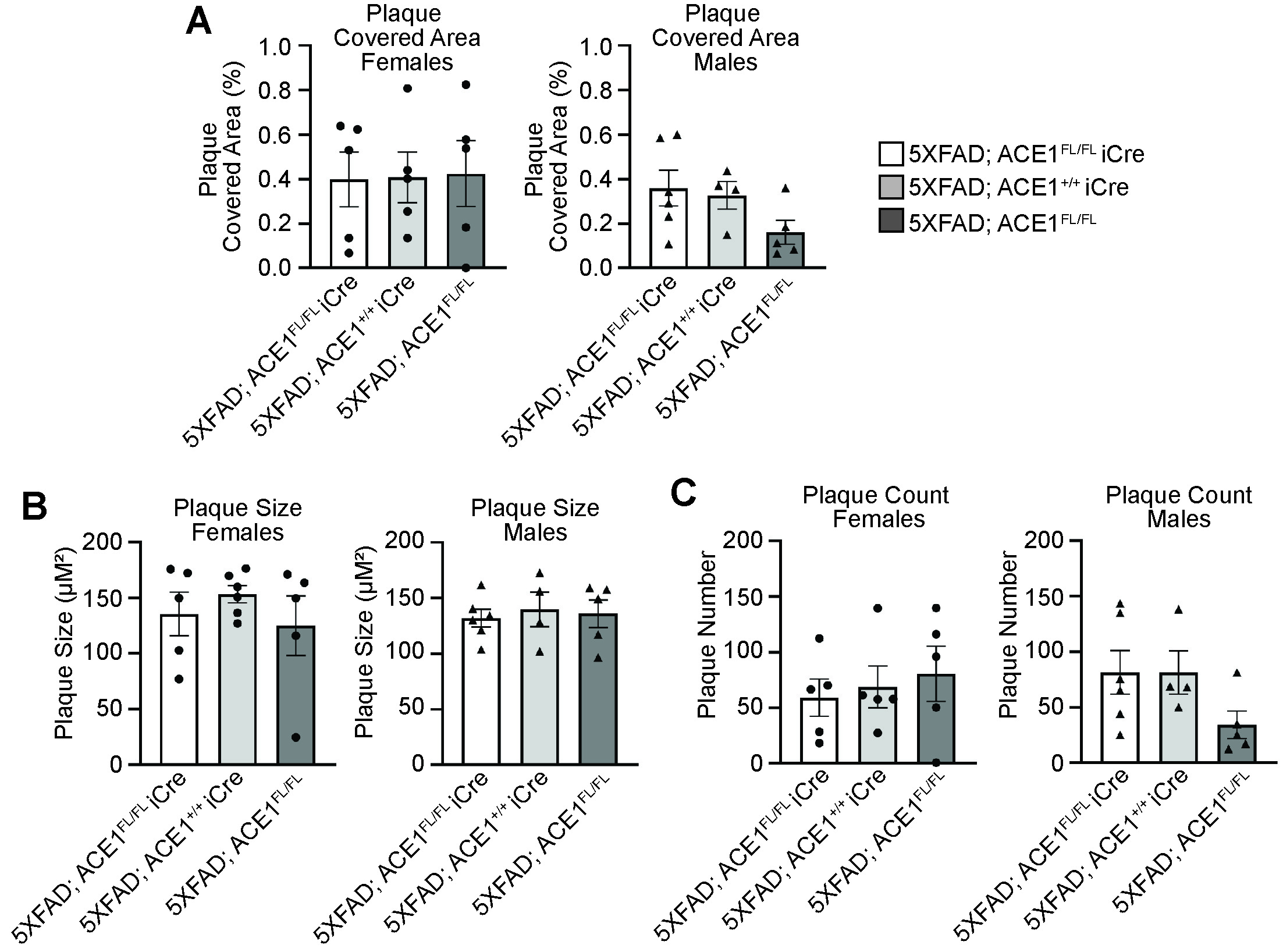

Supplement: S5 Fig — (A to C) Imaging analysis of coronal sections from the hippocampus of 6-months-old 5XFAD; ACE1FL/FLiCre, 5XFAD; ACE1+/+iCre, and 5XFAD; ACE1FL/FL mice. Quantification of plaque covered area (A), plaque size (B), and plaque count (C) in (Fig. 4) through independent analysis of females (left) and males (right). Females (5XFAD; ACE1FL/FLiCre, n = 5; 5XFAD; ACE1+/+iCre, n = 5–6; 5XFAD; ACE1FL/FL, n = 5). Males (5XFAD; ACE1FL/FLiCre, n = 6; 5XFAD; ACE1+/+iCre, n = 4; 5XFAD; ACE1FL/FL, n = 5). One-way ANOVA with Tukey’s multiple comparisons post hoc test was performed in (A to C). Circles represent data for female and triangles for male mice. *P < 0.05, **P < 0.01, ***P < 0.001, ****P < 0.0001. (TIF) [file pone.0330193.s003.tif]

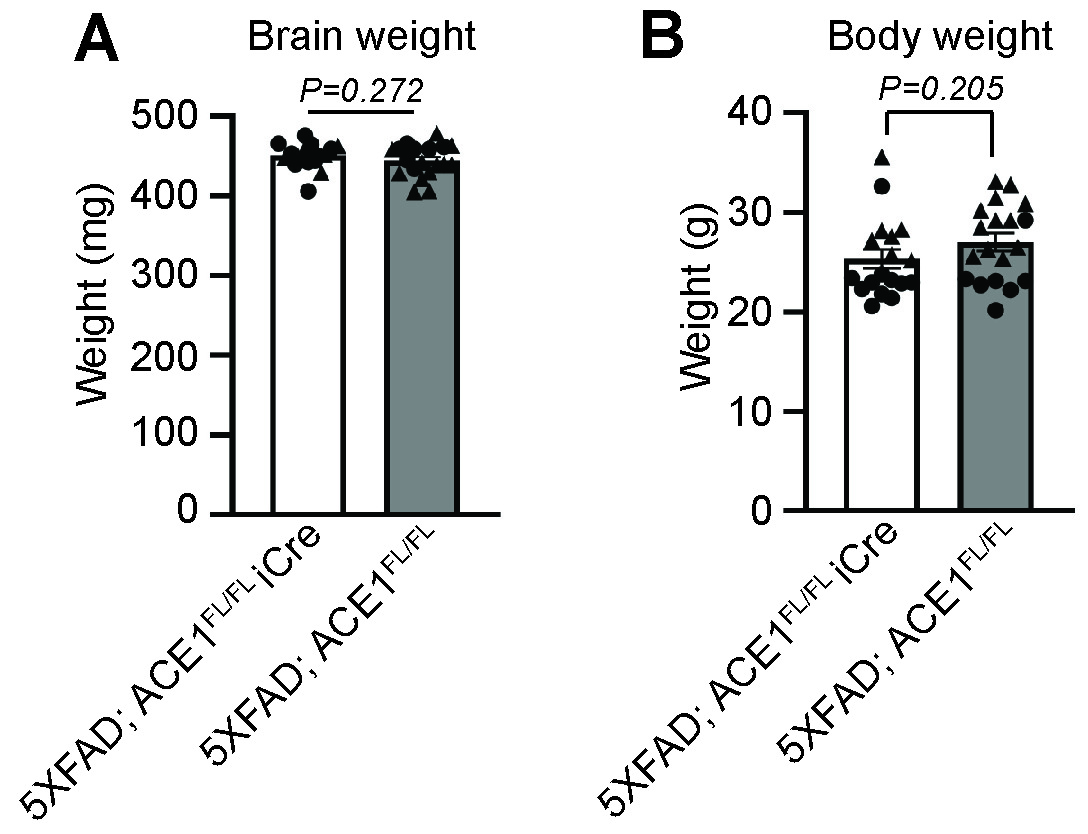

Supplement: S6 Fig — (A) Quantification of the brain weights (mg) from 6-months old 5XFAD; ACE1FL/FLiCre and 5XFAD; ACE1FL/FL mice. (5XFAD; ACE1FL/FLiCre, n = 18; 5XFAD; ACE1FL/FL, n = 19). (B) Quantification of the body weights (g) from 6-months old 5XFAD; ACE1FL/FLiCre and 5XFAD; ACE1FL/FL mice (5XFAD; ACE1FL/FLiCre, n = 18; 5XFAD; ACE1FL/FL, n = 19). Unpaired t test in (A to B). Circles represent data for female and triangles for male mice. *P < 0.05, **P < 0.01, ***P < 0.001, ****P < 0.0001. (TIF) [file pone.0330193.s004.tif]
